# His Tooth Exploaded

**Published:** 1895-06

**Authors:** 


### His Tooth Exploaded.

A short time ago William Arnold, an employe of the Carnegie mills at Beaver Falls, Pa., was attacked with a violent toothache in a big "double" molar. The man has a dread of dentists, and he could not be brought to the point of having it extracted. The pain grew into a paroxysm of agony, and while the man was writhing there was a muffled explosion in his mouth, and the next instant he was spitting blood and the fragments of the tooth from a badly lacerated mouth. Dentists say that the breaking of a diseased tooth in such paroxysms of pain is not unparalleled.—*Exchange.*

